# The presence of predators constrains larval development process by influencing critical developmental windows in the endangered Chinhai spiny newt

**DOI:** 10.1002/ece3.11396

**Published:** 2024-05-11

**Authors:** Xihong Zhu, Xia Qiu, Kaiyang Chen, Wei Li, Aichun Xu

**Affiliations:** ^1^ College of Life Sciences China Jiliang University Hangzhou China; ^2^ Zhejiang Museum of Natural History Hangzhou China

**Keywords:** critical developmental windows, *Echinotriton chinhaiensis*, endangered species, predator‐induced plasticity

## Abstract

Predators significantly impact the development process and subsequently influence the metamorphic decisions of amphibian larvae. Larvae often exhibit induced growth and metamorphic plasticity in response to the presence of predators. However, growth and development rates are not always perfectly correlated, growth responses can vary throughout ontogeny. It is crucial to consider the stage‐specific growth responses induced by predators. Here, we employ a critical windows experimental design and examine development‐related growth and metamorphic responses to predators in the endangered Chinhai spiny newt (*Echinotriton chinhaiensis*). Our findings reveal that predators constrain the development process of spiny newt larvae and also impact survival to metamorphosis. Inducible plasticity predominantly exhibits in the early and middle stages of larval development. Our results also suggest that diverse developmental plasticity has been adopted by larvae in response to predators. The presence of predators during early stage induces larvae to exhibit a same size at metamorphosis but a prolonged time to metamorphosis, while predators present during middle stage induce larvae to exhibit a large size at metamorphosis but a same time to metamorphosis. The presence of predators at the late developmental stage does not induce any plasticity in larval growth and metamorphosis. Moreover, these results also suggest that several stages of larval development are likely critical developmental windows for spiny newt larvae. This study not only provides basic biological information on predator‐induced developmental plasticity of the endangered Chinhai spiny newt but also likely provides biological insights for the implementation of in situ conservation and preservation efforts for endangered species.

## Graphical abstract

Our research highlights predator‐induced plasticity in the development of the endangered Chinhai spiny newt, revealing a dynamic metamorphic response to predators, with larvae in different stages likely exhibiting distinct predator‐induced plasticity. Specifically, our study demonstrates that predators constrain the development of spiny newt larvae, particularly during the early and middle stages, emphasizing these stages as crucial developmental windows for predator‐induced plasticity. Meanwhile, the late stage likely serves as developmental windows that limit such plasticity.

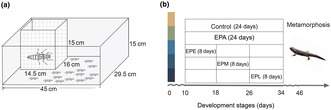

## 1 INTRODUCTION

Metamorphic decision induced by predators is an essential topic in understanding the complex life history of amphibians. Predators significantly impact the development process in larval amphibians, resulting in the following two metamorphic outcomes. Firstly, many studies have shown that the majority of larvae may be induced at the same or later time to metamorphosis in response to the presence of predators (Benard, 2004; Relyea, 2007). For example, tadpoles of the red legged frog, *Rana aurora*, increase their larval time and size at metamorphosis in response to the presence of predators (Barnett & Richardson, 2002). Secondly, a few studies suggest that larvae may be induced earlier time to metamorphosis in response to the presence of predators (Benard, 2004; Relyea, 2007). For example, tadpoles of *Bufo bufo* (Lardner, 2000) and *Hyla intermedia* (Sergio et al., 2021) reduce the time to metamorphosis, and exhibit a smaller or same size at metamorphosis.

The predator‐induced growth response is a significant factor for larval amphibians, affecting their metamorphic decisions. Some non‐exclusive theoretical models predict the relationship between growth and metamorphic decisions. Firstly, Wilbur and Collins' model proposes that the current growth rate determines the time to (and consequently the size at) metamorphosis. This model predicts that when faced with the risk posed by predators, larvae tend to metamorphose earlier (Wilbur & Collins, 1973). Secondly, Smith‐Gill and Berven's model proposes while growth rate and developmental stage differentiation are often correlated, the developmental rate more reliably predicts the time to metamorphosis (Smith‐Gill & Berven, 1979). Thirdly, Werner and Gilliam's model proposes that both mortality rate and growth rate determine the time to metamorphosis. This model predicts that when faced with the risk posed by predators, larvae tend to balance the benefits of growth against the risk of mortality during metamorphosis. Based on these models, a substantial amount of experimental research has investigated the effects of predator‐induced plasticity responses on metamorphic decisions over the past few decades (Benard, 2004; Relyea, 2007).

In general, the majority of amphibian larvae (e.g., *Triturus alpestris*, *R. aurora*, *R. sphenocephala*) exhibited the same or later time to metamorphosis in response to the presence of predators. This is attributed to the direct or indirect predator‐induced slowing growth and developmental rates (Babbitt, 2001; Barnett & Richardson, 2002; Van Buskirk & Schmidt, 2000). However, growth and development rates are not always perfectly correlated; growth responses can vary throughout ontogeny (Relyea, 2003; Smith‐Gill & Berven, 1979). It is crucial to consider the stage‐specific growth responses induced by predators. Consequently, there may be critical developmental windows, periods during which an organism is particularly plastic to the presence of predators (Mueller, 2018). For example, the gray treefrog tadpoles (*H. versicolor*) easily changed their plasticity phenotype responses to the presence of predators over ontogeny. However, the differences in plasticity responses were particularly pronounced between early and later ontogeny stages, suggesting that there were few developmental windows to restrict predator‐induced responses (Relyea, 2003). Determining whether there are specific developmental critical windows in which preys show increased sensitivity to the presence of predators can enrich the plasticity and metamorphic decision theories. Additionally, it may provide biological information for the implementation of in situ conservation and preservation efforts for endangered species.

The Chinhai spiny newt, *Echinotriton chinhaiensis*, is among the critically endangered salamander species, with limited numbers found in the eastern part of Zhejiang province, China. Since 2004, it has been classified as “Critically endangered” on the IUCN Red List of Threatened Species due to the rarity of natural populations (Xie & Gu, 2004). Predators pressure stands out as a key factor contributing to the endangered status of the Chinhai spiny newt (Xu et al., 2021). The early development of the Chinhai spiny newt, from fertilization to metamorphosis completion, lasts for approximately 120 days (Xie et al., 2001). The aquatic larval stage, constituting three‐fourths of the entire early development (prior to metamorphosis) of the Chinhai spiny newt, lasts 58–88 days (Xie et al., 2001). The suitable habitat for these larvae is very specialized and limited, still‐water ponds located at the border of agricultural and forested areas, with sizes at approximately two square meters. These ponds harbor a substantial population of dragonfly nymphs, estimated at around five individuals per square meter (Results of field observations). Given the confined size of the ponds, dragonfly nymphs present a significant predator risk to the Chinhai spiny newt larvae. Therefore, investigating the plasticity of the Chinhai spiny newt during its larval developmental stage in response to predators, especially the growth responses associated with developmental critical windows, enriches the basic biological information of this endangered species. It may also play some role in the in situ conservation of the Chinhai spiny newt.

Here, we employ a critical windows experimental design to examine predator‐induced development‐related responses in the Chinhai spiny newt. Our objectives are to (1) examine whether the presence of predators influences the growth and metamorphic traits of the Chinhai spiny newt larvae; (2) determine whether the impacts of predators on the growth and metamorphic traits in the Chinhai spiny newt larvae are associated with their developmental stages; (3) identify any critical developmental windows for plastic responses to the presence of predators. We predict (1) the Chinhai spiny newt larvae will respond to the presence of predators by exhibiting slower growth and will be induced to metamorphose at a same or a later time; (2) the larvae will exhibit changes in their predator‐induced plasticity over developmental stages, while the plasticity in late developmental stage will be restricted; (3) there are critical developmental windows that plastic or restrict predator‐induced responses.

## 2 MATERIALS AND METHODS

### 2.1 Animal collection and maintenance

During late April to early May 2022, we collected 15 clutches of freshly laid eggs of the Chinhai spiny newt from the Ruiyansi Forest Park (29°48′24″ N, 121°51′12″ E) in the Ningbo municipality, China. Eggs were transported to our laboratory located within the park, where these eggs were maintained in their respective clutches until hatching. The hatching process was completed under natural photoperiod and temperature conditions in a cylindrical plastic tank (7.5 cm high and 20 cm in diameter). In each hatching tank, 1.5 centimeters of water was added and a regular water‐absorbing sponge (17 × 12 × 2 cm) was placed in the water, ensuring that it remained in full contact with the water to maintain the humidity required for egg hatching. The larvae were reared in several rectangular plastic tanks (45 × 29.5 × 14.5 cm) with 4.5 cm of water. All water used in the experiments was collected from the ponds where the eggs were collected.

Dragonfly larvae (*Polycanthagyna ornithocephala*), a native predator of the Chinhai spiny newt larvae, were collected from the ponds near the egg collection location. To avoid size‐related effects, all selected dragonfly larvae were individuals of similar size (length: mean ± SD = 4.64 ± 0.18 cm, mass: mean ± SD = 1.47 ± 0.09 g) in the late‐instar stage. Dragonfly larvae were maintained in a plastic tank (85 × 56 × 18 cm) with aquatic plants and water from the collection location. Aquatic plants were placed to provide refugia for the dragonfly larvae. We fed tubifex worms to predators once a day before the experiments.

### 2.2 Experiment design

**FIGURE 1:**
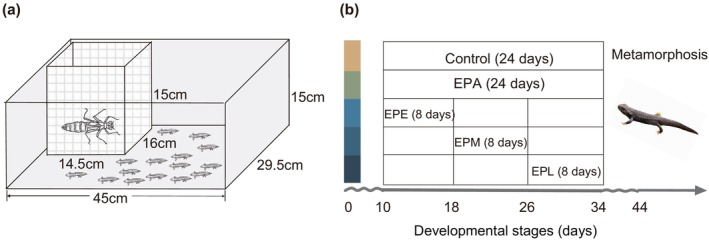
Schematic diagram illustrating (a) the simulation of predator conditions for spiny newt larvae and (b) The critical windows experimental design, with color squares representing corresponding experimental treatments. In total, there were five experimental treatments: spiny newt larvae were exposed to predator conditions during all developmental stages (EPA), spiny newt larvae were exposed to predator conditions only during the early developmental stage (EPE), spiny newt larvae were exposed to predator conditions only during the middle developmental stage (EPM), spiny newt larvae were exposed to predator conditions only during the late developmental stage (EPL), and spiny newt larvae were not exposed to predator conditions during any developmental stages as the control group (Control).

We simulated predator conditions and used critical windows experimental design to evaluate the growth and metamorphic responses of the Chinhai spiny newt larvae to the presence of predators. A net cage (14.5 × 16 × 15 cm, mesh size: 1 mm) containing the dragonfly larvae was placed inside the rearing tank of the newt larvae to simulate a predator condition (Figure 1a). The net cage allowed cues to be transmitted but did not allow predation. Previous studies have indicated that cues from both predators and preys could trigger predator‐induced response in prey (Anderson & Mathis, 2016; Kłosiński et al., 2022; Lucon‐Xiccato et al., 2018). Therefore, we assumed that this designed predator condition would create a predator threat through predator cues and prey alarm cues. Due to the endangered status of the Chinhai spiny newt, the newt larvae were not used as food for dragonfly larvae to stimulate the release of predator cues by the predators, although this might have decreased the size of the predator‐induced effect. Instead, we collected another prey of the dragonfly larva, tadpoles of the Zhenghai wood frog (*R. zhenhaiensis*), which coexist with newt larvae in the same ponds. These tadpoles were used as food during experiments for the dragonfly larvae to stimulate the release of predator cues by the predators. At the same time, the wood frog tadpoles likely released alarm cues, which might also alert the spiny newt larvae.

We exposed the spiny newt larvae to the predator condition separately during each of their three developmental stages (the critical window design). The three stages were divided according to the developmental curve of this species (Xie et al., 2001). The early developmental stage includes the period from the 10th day after hatching to the 18th day, during which the first four toes on hind limbs develop. The middle developmental stage includes the period from the 18th day after hatching to the 26th day, during which the fifth toe on the hind limbs develops. The late developmental stage includes the period from the 26th day after hatching to the 34th day, during which the external gill atrophy occurs. Therefore, we designed five experimental treatments (Figure 1b), including (1) spiny newt larvae were exposed to predator conditions during all developmental stages (EPA), (2) spiny newt larvae were exposed to predator conditions only during the early developmental stage (EPE), (3) spiny newt larvae were exposed to predator conditions only during the middle developmental stage (EPM), (4) spiny newt larvae were exposed to predator conditions only during the late developmental stage (EPL), and (5) spiny newt larvae were not exposed to predator conditions during any developmental stages as the control group (Control). By comparing the responses on growth and metamorphic traits of the Chinhai spiny newts between the control group and the EPA group, we would determine whether the predators had an effect on the newts. By comparing the responses between the control group and the other experimental groups, we would determine whether there was a critical development window and would identify the window(s).

### 2.3 Experiment procedure

#### 2.3.1 Experimental groups assignment

Twenty newly hatched spiny newt larvae from each of the 15 sampled clutches, hatched within the first 5 days, were randomly assigned into 20 rearing tanks to ensure that the maximum age difference within each tank did not exceed 5 days. Each rearing tank contained 15 individuals, which were sourced from 15 different clutches. This arrangement helped to ensure that the genetic variation among the rearing tanks was equivalent. We randomly assigned the 20 rearing tanks to the five experimental treatments, with four tanks in each group. After the assignment, 10 days were given to acclimate to the rearing environment. We did not feed the larvae during this acclimation period. The water temperature ranged from 21 to 23°C during the rearing period.

The predator conditions were initiated after 10 days of acclimation. Dragonfly larvae were placed in the net cage according to the order of each experimental group. A total of 16 dragonfly larvae were randomly placed in each tank of the experimental groups requiring predator exposure. The net cages were kept empty for the four tanks of the control group. Dragonfly larvae were fed wood frog tadpoles once a day during this period to ensure they released sufficient predator cues. Spiny newt larvae were fed brine shrimp (3 mL) three times a day to ensure sufficient food for them. The rearing water was replaced every day, and the temperature ranged from 21 to 23°C during the rearing period.

#### 2.3.2 Measurements of growth and metamorphic traits

For growth rate, we measured body length and body mass of each spiny newt larva during all predator condition‐exposure periods, with the longest exposure treatment period being 24 days. We were unable to tag individuals to track the body length and body mass of the same individual at different developmental stages. Measuring times were at the beginning of the predator condition experiment (the 10th day), and then at 8‐day intervals until 34 days when the stimuli were removed. Body length was defined as the distance from the snout to the tip of the tail in a spiny newt larva. Each individual was photographed using a camera (Company Zhongweikechuang, type ZW‐U500) controlled by S‐EYE software (version 1.4.4.500), with a 0.5 cm scale bar included in the photos for length calibration. Body length was taken from the photos using Image J software (version 1.53e) to the nearest 0.001 cm. Body mass was measured to the nearest 0.0001 g using an electronic balance (G&G JJ224BF), after quickly blotting the larvae using an absorbent paper. Upon completing the measurements, all individuals were returned to their original rearing tank.

For metamorphic traits, we measured body length and mass at metamorphosis, time to metamorphosis, and survival to metamorphosis. When an individual comes ashore and loses the external gills, it is considered to have completed metamorphosis (Xie et al., 2001). On this day, we measured the body length and mass of each individual as metamorphic traits, using the same measurement methods as those used for growth measurements. Time to metamorphosis was defined as the time duration of the spiny newt larva from hatching to metamorphosis for each spiny newt larva. Survival to metamorphosis was defined as the proportion of spiny newt larvae that successfully reached metamorphosis. Any individuals that did not metamorphose all died during the process of reaching metamorphosis.

### 2.4 Statistical analysis

We used linear mixed models and generalized mixed models in R package *lme4* (Bates et al., 2015; R Core Team, 2023) to examine the variation in growth and metamorphic measurements of the Chinhai spiny newt larvae. For growth rate, we considered body length and mass separately as a dependent variable, with treatments (Control, EPA, EPE, EPM, and EPL), larval developmental stages, and the interaction of treatments and larval developmental stages as fixed effects, while assuming a Gaussian error distribution. To account for the variance of within‐treatment, we treated replicates as a random effect. The significance of models was assessed with the “*anova*” function applying type III sums of squares calculations to test interaction terms. Then we separately compared the variation in body length and body mass at different developmental stages between predator treatments and control group.

The predator condition‐exposure treatments (EPA, EPE, EPM, and EPL) each had one replicate experimental group in which the predator died during the experiment (after the 26th day), resulting in only three replicates available for the statistical analysis of metamorphic measurements in the end. For metamorphic traits, we performed the same analysis for body length and body mass measured at metamorphosis. For time to metamorphosis, we performed a similar analysis but assumed a Poisson error distribution. We also performed a similar analysis for survival to metamorphosis, assuming a Binomial distribution, treating survival as binary dependent variable, with “1” representing survival, while “0” representing died.

We use the R package *lmerTest* to assess the significance of factors (Kuznetsova et al., 2017). The normality of model residuals was tested using “*qqnorm*” function in the R base package stats and used as a measure of model goodness of fit. We used *ggplot2* package in R to visualize the growth and metamorphic variation between treatments (Wickham, 2010).

## 3 RESULTS

### 3.1 Growth response

Both body length and body mass were significant for the interaction between developmental stages and treatment (Table 1). On the first day of the predator treatment, which corresponds to the 10th day of the spiny newt larval developmental stage, we did not find any significant differences in both body length and body mass between the control group and predator treatment groups (Tables 2 and 3). On the 18th day of the spiny newt larval developmental stage, the body mass of larvae in both the EPA group (estimate = −0.015, *t* = −3.204, *p* = .006) and the EPE group (estimate = −0.014, *t* = −3.185, *p* = .007) were significantly lighter than those in the control group. No significant difference in body mass was detected between the control group and other treatment groups (EPM and EPL). Additionally, no significant difference in body length was detected between the control group and the predator treatment groups (Tables 2 and 3). On the 26th day of the spiny newt larval developmental stage, we found that the larvae in the EPM group were significantly shorter (estimate = −0.240, *t* = −3.665, *p* = .006) and lighter (estimate = −0.065, *t* = −3.835, *p* = .004) than those in the control group. No significant difference in either body length or body mass was detected between the control group and other predator treatment groups (EPA, EPE, and EPL, Tables 2 and 3). On the 34th day of the spiny newt larval developmental stage, we found that larvae in the EPM group were significantly lighter (estimate = −0.056, *t* = −2.971, *p* = .016) than those in the control group, and no significant difference in body mass was detected between the control group and other predator treatment groups (EPA, EPE, and EPL, Tables 2 and 3). No significant difference in body length was detected between the control group and the predator treatment groups (EPA, EPE, EPM, and EPL, Tables 2 and 3).

**TABLE 1:** Summary of ANOVA examining the growth variations of larval Chinhai spiny newts among developmental stages, treatments, and the interaction of developmental stages and treatments.

Fixed effects	Sum Sq	Mean Sq	Num df	Den df	*F* value	*p* value
Body length						
Treatments	0.179	0.045	4	15.95	1.325	.303
**Developmental stages**	**256.808**	**85.603**	**3**	**866.08**	**2531.607**	**<.0001**
**Treatments: developmental stages**	**4.976**	**0.415**	**12**	**865.20**	**12.264**	**<.0001**
Body mass						
**Treatments**	**0.031**	**0.008**	**4**	**16.13**	**7.223**	**.002**
**Developmental stages**	**9.108**	**3.036**	**3**	**867.19**	**2832.691**	**<.0001**
**Treatments: Developmental stages**	**0.140**	**0.012**	**12**	**865.56**	**10.870**	**<.0001**

*Note*: Significant differences (*p* < .05) are highlighted in bold.

**TABLE 2:** Comparisons of means and standard deviations for the growth and metamorphic traits of larval Chinhai spiny newts were conducted between the predator treatment groups and the control group at various developmental stages.

Measurements	Control group	Predator treatment groups			
		EPA	EPE	EPM	EPL
Growth rate					
The 10th day	*N* = 60	*N* = 60	*N* = 60	*N* = 60	*N* = 60
Body length (cm)	1.92 ± 0.09	1.92 ± 0.1	1.91 ± 0.1	1.93 ± 0.1	1.92 ± 0.11
Body mass (g)	0.05 ± 0.01	0.04 ± 0.01	0.04 ± 0.01	0.04 ± 0.01	0.04 ± 0.01
The 18th day	*N* = 51	*N* = 33	*N* = 41	*N* = 59	*N* = 50
Body length (cm)	1.91 ± 0.26	2.08 ± 0.15	2.04 ± 0.2	2.09 ± 0.2	1.86 ± 0.26
Body mass (g)	0.09 ± 0.02	**0.07 ± 0.01**	**0.07 ± 0.02**	0.08 ± 0.01	0.08 ± 0.01
The 26th day	*N* = 49	*N* = 30	*N* = 29	*N* = 44	*N* = 32
Body length (cm)	2.84 ± 0.2	2.77 ± 0.24	2.79 ± 0.28	**2.6 ± 0.23**	2.88 ± 0.22
Body mass (g)	0.21 ± 0.04	0.17 ± 0.04	0.18 ± 0.05	**0.14 ± 0.03**	0.2 ± 0.04
The 34th day	*N* = 49	*N* = 26	*N* = 26	*N* = 43	*N* = 31
Body length (cm)	3.29 ± 0.18	3.37 ± 0.24	3.4 ± 0.29	3.14 ± 0.24	3.39 ± 0.22
Body mass (g)	0.33 ± 0.05	0.33 ± 0.06	0.36 ± 0.07	**0.28 ± 0.06**	0.33 ± 0.06
Metamorphic traits					
Length at metamorphosis (cm)	3.57 ± 0.23	**3.85 ± 0.15**	**3.86 ± 0.25**	3.57 ± 0.2	3.72 ± 0.23
Mass at metamorphosis (g)	0.38 ± 0.05	**0.42 ± 0.05**	**0.44 ± 0.08**	0.36 ± 0.06	0.4 ± 0.06
Time to metamorphosis (day)	41 ± 4	**45 ± 2**	44 ± 3	**45 ± 4**	44 ± 4
Survival to metamorphosis/Total	*N* = 41/60	*N* = 22/45	*N* = 19/45	*N* = 36/45	*N* = 25/45

*Note*: Significant differences (*p* < .05) between the predator treatment groups and control group are highlighted in bold.

**TABLE 3:** Summary of linear mixed models examining the difference in the body length and mass of larval Chinhai spiny newts between the predator treatment groups and the control group at various developmental stages.

Developmental stages	Measurements	Compared to the control group	Estimate	SE	*t* value	*p* value
The 10th day	Body length	EPA	−0.004	0.018	−0.209	.835
		EPE	−0.015	0.018	−0.834	.405
		EPM	0.009	0.018	0.490	.625
		EPL	−0.004	0.018	−0.209	.835
	Body mass	EPA	−0.001	0.001	−0.468	.640
		EPE	−0.001	0.001	−0.937	.349
		EPM	−0.000	0.001	−0.274	.784
		EPL	−0.001	0.001	−0.785	.433
The 18th day	Body length	EPA	0.114	0.127	0.896	.384
		EPE	0.113	0.125	0.907	.379
		EPM	0.155	0.124	1.253	.230
		EPL	−0.045	0.124	−0.359	.725
	Body mass	**EPA**	**−0.015**	**0.005**	**−3.204**	**.006**
		**EPE**	**−0.014**	**0.004**	**−3.185**	**.007**
		EPM	−0.009	0.004	−2.120	.056
		EPL	−0.002	0.004	−0.558	.587
The 26th day	Body length	EPA	−0.070	0.068	−1.033	.323
		EPE	−0.046	0.070	−0.654	.527
		**EPM**	**−0.240**	**0.065**	**−3.665**	**.006**
		EPL	0.038	0.069	0.554	.592
	Body mass	EPA	−0.035	0.017	−2.087	.060
		EPE	−0.025	0.017	−1.453	.175
		**EPM**	**−0.065**	**0.017**	**−3.835**	**.004**
		EPL	−0.004	0.017	−0.228	.824
The 34th day	Body length	EPA	0.071	0.080	0.887	.393
		EPE	0.114	0.080	1.421	.181
		EPM	−0.158	0.076	−2.091	.065
		EPL	0.100	0.078	1.280	.227
	Body mass	EPA	−0.008	0.020	−0.379	.712
		EPE	0.025	0.020	1.239	.240
		**EPM**	**−0.056**	**0.019**	**−2.971**	**.016**
		EPL	−0.006	0.019	−0.322	.754

*Note*: Significant differences (*p* < .05) between the predator treatment groups and control group are highlighted in bold.

### 3.2 Metamorphic traits response

The metamorphic individuals in both the EPA group (body length: estimate = 0.287, *t* = 3.716, *p* = .003; body mass: estimate = 0.045, *t* = 2.407, *p* = .037) and the EPE group (body length: estimate = 0.298, *t* = 3.771, *p* = .003; body mass: estimate = 0.067, *t* = 3.442, *p* = .005) were significantly longer and heavier than those in the control group (Figure 3a,b). No significant difference in either body length or body mass was detected between the control group and other treatment groups (EPM and EPL, Tables 2 and 4). In terms of time to metamorphosis, the individuals in both the EPA group (estimate = 0.091, *z* = 2.290, *p* = .022) and the EPM group (estimate = 0.090, *z* = 2.584, *p* = .010) took significantly more time to undergo metamorphosis than those in the control group (Figure 3c). No significant difference in metamorphic time was detected between the control group and other treatment groups (EPE and EPL, Tables 2 and 4).

**TABLE 4:** Summary of linear mixed models examining the difference in the metamorphic traits of larval Chinhai spiny newts between the predator treatment groups and the control group.

Metamorphosis traits	Compared to the control group	Estimate	SE	*t*/*z* value	*p* value
Length at metamorphosis	**EPA**	**0.287**	**0.077**	**3.716**	**.003**
	**EPE**	**0.298**	**0.079**	**3.771**	**.003**
	EPM	0.002	0.072	0.023	.982
	EPL	0.164	0.076	2.162	.056
Mass at metamorphosis	**EPA**	**0.045**	**0.019**	**2.407**	**.037**
	**EPE**	**0.067**	**0.020**	**3.442**	**.005**
	EPM	−0.020	0.017	−1.140	.293
	EPL	0.020	0.018	1.087	.306
Time to metamorphosis	**EPA**	**0.091**	**0.040**	**2.290**	**.022**
	EPE	0.064	0.042	1.517	.129
	**EPM**	**0.090**	**0.035**	**2.584**	**.010**
	EPL	0.059	0.039	1.529	.126
Survival to metamorphosis	**EPA**	**−0.814**	**0.407**	**−1.997**	**.046**
	**EPE**	**−1.083**	**0.410**	**−2.641**	**.008**
	EPM	0.617	0.465	1.328	.184
	EPL	−0.456	0.410	−1.111	.267

*Note*: Significant differences (*p* < .05) between the predator treatment groups and control group are highlighted in bold.

Additionally, the proportion survival to metamorphosis was significantly lower in both the EPA group (estimate = −0.814, *z* = −1.997, *p* = .046) and EPE group (estimate = −1.083, *z* = −2.641, *p* = .008) compared to the control group (Figure 3d). No significant difference in proportion survival to metamorphosis was detected between the control group and other treatment groups (EPM and EPL, Tables 2 and 4).

## 4 DISCUSSION

Our research offers insights into the development‐related predator‐induced plasticity of the endangered Chinhai spiny newt. The presence of predators clearly constrains the development process of spiny newt larvae and impacts their survival to metamorphosis. Furthermore, these predator‐induced growth and metamorphic responses are primarily observed in the early and middle stages of larval development. Moreover, predator‐induced metamorphic plasticity varies across different developmental stages. The presence of predators during the early stage induced larvae to exhibit the same size at metamorphosis but prolong time to metamorphosis, while predators present during the middle stage induced larvae to exhibit a large size at metamorphosis but same time to metamorphosis. The presence of predators at the late developmental stage did not induce any plasticity in the growth and metamorphosis of larvae. These results suggest that several stages of larval development are likely critical developmental windows for spiny newt larvae.

**FIGURE 2:**
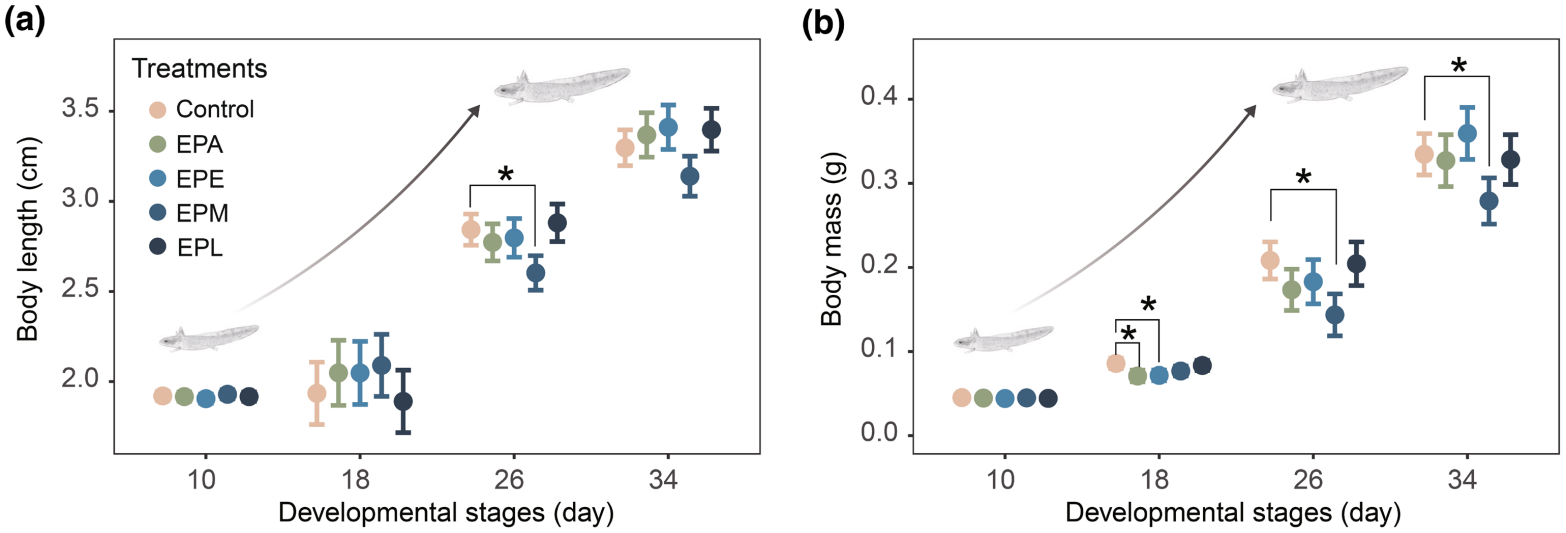
Effects of experimental treatments at different developmental stages on (a) the body length and (b) body mass of the larval Chinhai spiny newts. Colorful points are the predicted average value, and error bars represent the 95% confidence interval. “*” indicates significant variation.

**FIGURE 3:**
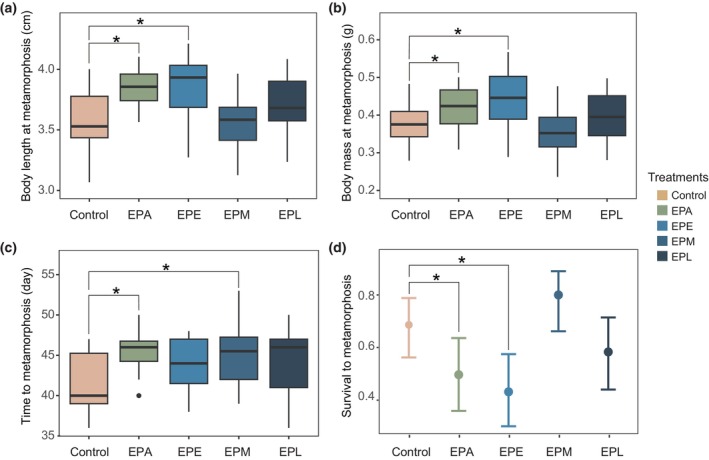
Effects of experimental treatments on metamorphic traits of the larval Chinhai spiny newts. Box plots show differences in (a) Body length and (b) body mass at metamorphosis, (c) time to metamorphosis between predator treatment groups and control group. Black points in the figure show the outliers of conspicuousness. (d) Comparisons for survival to metamorphosis between predator treatment groups and control group. Colorful points are the predicted average value, and error bars represent the 95% confidence interval. “*” indicates significant variation.

We demonstrate the presence of predators constrains the development process of spiny newt larvae and also impacts survival to metamorphosis. The results of our experiments on growth and metamorphic responses strongly support this conclusion. Firstly, compared to the control group with the absence of predators, the larval growth rate was significantly slowed down after 8 days of the presence of predators (Figure 2, EPA, EPE, and EPM groups). Secondly, under the presence of predators, spiny newt larvae underwent a prolonged time to metamorphosis (Figure 3c, EPA and EPM groups), and exhibited a larger size at metamorphosis (Figure 3a,b, EPA and EPE groups) compared to the absence of predators, highlighting a significant impact of the predators on metamorphic decisions. Developmental plasticity is a common response when facing caged predators in other larval salamanders, for instance *Ambystoma opacum* and *T. alpestris* (Davenport & Chalcraft, 2014; Van Buskirk & Schmidt, 2000). One possible explanation is that predator cues can increase metabolic and physiological costs, which mediate the growth and developmental restriction (Joshi et al., 2017; Steiner, 2007). For example, in *R. lessona*, physiological mechanisms such as intestinal emptying influence growth rate (Steiner, 2007). Lastly, the presence of predators reduced the proportion of survival to metamorphosis of larvae (Figure 3d), significantly influencing larval metamorphic capability. All these results highlight the crucial role of predators in the developmental process of spiny newt larvae.

Our results also reveal that larval Chinhai spiny newts are induced in different plasticity across different developmental stages. Compared to the absence of predators, when spiny newt larvae were exposed to predators only during the early developmental stage, they reached their metamorphosis with larger body sizes but spent a same time to metamorphosis (Figure 3). Here, the larval Chinhai spiny newts likely accelerate growth rate in later stages to be large at metamorphosis. This is evidenced by slower growth observed during this stage (Figure 2) and similar metamorphic times between larvae in the EPE group and the control group (Tables 2, 3 and 4). When spiny newt larvae were exposed to predators during the middle developmental stage, they slowed down the growth at this stage, but also exhibited reduced growth in the later stage when predators were absent. Consequently, the larvae underwent a prolonged time to metamorphosis but remained the same size. The prolonged time to metamorphosis likely explains why metamorphic survival was not affected, as there exists a minimum size requirement for successful metamorphosis (Berec et al., 2016; Gilbert et al., 2020). Here, the prolonged metamorphic time but same body size may fulfill the minimum requirements for metamorphosis. Previous studies have reported similar results, indicating that the presence of predators leads to a longer or same time to metamorphosis and a larger or same size at metamorphosis in other larval salamanders, such as *Euphlyctis cyanophlyctis* (Supekar & Gramapurohit, 2020) and *T. alpestris* (Van Buskirk & Schmidt, 2000). During the subsequent terrestrial stage, a larger body size may reduce predator palatability, thereby enhancing the ability to cope with predation risk and improve survival (Laurila & Kujasalo, 1999; Lent & Babbitt, 2020).

Our observations suggest there are likely several developmental windows in spiny newt larvae. Firstly, both the early and middle stages likely serve as developmental windows for predator‐induced plasticity, while the late stage likely serves as developmental windows that restrict plasticity. This is because the larvae exhibited slower growth in response to the predator present during the early and middle developmental stages (Figure 2). However, the larvae did not display this growth response when predator was present in the late developmental stage (Figure 2). Secondly, the early stages likely serve as developmental windows that restrict larval survival (Figure 3d). This is evidenced by the slower proportion survival rate of larvae to metamorphosis in response to the predator present during the early developmental stage (Figure 3). The earlier developmental stages as critical developmental windows for inducible plasticity have been observed in multiple amphibians, but it is proposed that inducible plasticity is commonly stronger during early embryonic development than during larval development (Lehman & Campbell, 2007; Mandrillon & Saglio, 2008; Shaffery & Relyea, 2015). Our results enrich the insights into predator‐induced critical developmental windows in early larval development. However, it should be noted that the developmental windows for predator‐induced plasticity in spiny newts may occur earlier than the period we observed in our experiments. This is because there is a 10‐day period in the earliest stage of spiny newt development, which corresponds to nearly one‐fifth of their larval period (10/44), that falls within the acclimation of our study.

In conclusion, our study reveals that predators exert constraints on the development process of spiny newt larvae, with potential implications for their survival. The inducible plasticity primarily occurs during the early and middle stages. This highlights the significance of the early and middle stages of larval development as developmental windows, where spiny newt larvae are particularly plastic to the presence of predators. While the late stage likely serves as developmental windows that restrict the plasticity. Furthermore, our observations suggest a dynamic response to predators, with larvae in different stages likely adopting distinct predator‐induced plasticity. Understanding the predator‐induced plasticity of the Chinhai spiny newt enriches not only the plasticity and metamorphic decisions theories but enhances the basic biological information of this endangered species. It provides roles in developing effective conservation strategies for the Chinhai spiny newt.

## AUTHOR CONTRIBUTIONS

**Xihong Zhu:** Data curation (lead); formal analysis (equal); methodology (equal); visualization (equal); writing – original draft (equal). **Xia Qiu:** Formal analysis (equal); visualization (equal); writing – original draft (equal); writing – review and editing (equal). **Kaiyang Chen:** Conceptualization (equal); methodology (equal). **Wei Li:** Data curation (equal); methodology (equal). **Aichun Xu:** Conceptualization (lead); funding acquisition (lead); writing – review and editing (equal).

## CONFLICT OF INTEREST STATEMENT

The authors declare no conflicts of interest.

## Data Availability

The datasets generated and analyzed during the current study are available on Science DB at https://doi.org/10.57760/sciencedb.14288.
